# Epiphytic bryozoans on Neptune grass – a sample-based data set

**DOI:** 10.3897/zookeys.606.8238

**Published:** 2016-07-21

**Authors:** Gilles Lepoint, André Heughebaert, Loïc N. Michel

**Affiliations:** 1 Laboratory of Oceanology, FOCUS research unit, University of Liège, Liège, Belgium; 2 Belgian Biodiversity Platform, Belgian Science Policy Office (BELSPO), Brussels, Belgium

**Keywords:** Epiphytism, biofouling, seagrass, Bryozoa, biodiversity, sample-based data set, ecological traits, Mediterranean Sea

## Abstract

**Background:**

The seagrass *Posidonia oceanica* L. Delile, commonly known as Neptune grass, is an endemic species of the Mediterranean Sea. It hosts a distinctive and diverse epiphytic community, dominated by various macroalgal and animal organisms. Mediterranean bryozoans have been extensively studied but quantitative data assessing temporal and spatial variability have rarely been documented. In Lepoint et al. (2014a, b) occurrence and abundance data of epiphytic bryozoan communities on leaves of *Posidonia oceanica* inhabiting Revellata Bay (Corsica, Mediterranean Sea) were reported and trophic ecology of *Electra posidoniae* Gautier assessed.

**New information:**

Here, metadata information is provided on the data set discussed in Lepoint et al. (2014a) and published on the GBIF portal as a sampling-event data set: http://ipt.biodiversity.be/resource?r=ulg_bryozoa&v=1.0).

The data set is enriched by data concerning species settled on *Posidonia* scales (dead petiole of *Posidonia* leaves, remaining after limb abscission).

## Introduction

In the marine environment, the term “epiphyte” is defined as: “all micro- or macro-organism living attached on a plant substrate” (Steel and Bastow Wilson 2003). The seagrass *Posidonia oceanica* L. Delile, commonly known as Neptune grass, is an endemic species of the Mediterranean Sea that hosts a distinctive and diverse epiphytic community, dominated by various macroalgal and animal organisms (Boero et al. 1985; Mazzella et al. 1989; Peres and Picard 1964; Van Der Ben 1971). Among epiphytic animals fixed on seagrass substrates, cheilostome bryozoans are often the most abundant and diverse taxa (Balata et al. 2007; Lepoint et al. 1999; Nesti et al. 2009; Pardi et al. 2006). Some species, like *Electra posidoniae* Gautier, are strictly found on Neptune grass leaves (Gautier 1961; Lepoint et al. 2014b; Matricardi et al. 1991). Due to this important contribution, cheilostome bryozoan diversity has received particular attention (Balduzzi et al. 1983; Gautier 1961; Harmelin 1973; Hayward 1975; Kocak et al. 2002; Lepoint et al. 2014a), but quantitative data are often lacking and seasonal variability is less often documented than spatial variability. This spatio-temporal variability was assessed quantitatively in Lepoint et al. (2014a) and the occurrence and abundance data set resulting from this study was made freely available on the GBIF portal as a sample-based data set. Biological and ecological features of the leaves’ community are also presented and discussed in Lepoint et al. (2014a). Trophic ecology of *Electra posidoniae*, an obligate epiphyte of *Posidonia oceanica*, are discussed in Lepoint et al. (2014b). Scale community data were only published in the GBIF data set but not discussed.

## General description of the dataset

The data set is a sample-based data set (n = 103 sampling events), recording occurrence (n = 1234) and abundance of cheilostome Bryozoa (n = 6488 counted colonies) settled as epiphytes on leaves and scales of the seagrass *Posidonia oceanica*. Sampling encompasses an entire annual cycle (from November 2002 to December 2003) (n = 5 sampling seasons) and six sampling depths (7, 10, 15, 20, 25, 30 m). The data set package is composed of two data files: the former describing sampling events and the latter reporting occurrence and abundance of bryozoan colonies.

## Project description

The data were collected during a postdoctoral project (G.L.) entitled: ”Nitrogen dynamics and ecology of the epiphyte community in a *Posidonia oceanica* seagrass bed”.

The *Posidonia* seagrass beds are one of the most important ecosystems in the Mediterranean coastal zone. Seagrass beds are in regression in many areas of the world, while the reasons of such regression are complex and often poorly understood. The epiphyte community constitutes an important component of the seagrass meadow. Variability in epiphyte community composition and/or biomass may sometimes be linked to anthropogenic disturbance and used as monitoring tool (Mabrouk et al. 2013; Martinez-Crego et al. 2010; Piazzi et al. 2004). This argues for more fundamental studies of the seagrass ecosystem functioning and epiphyte biodiversity.

The general objective of this project was to study the dynamics of nitrogen in the epiphyte community of the *Posidonia* leaves in relation with its ecology and its spatio-temporal structure.

Sampling and experiments were performed in Revellata Bay (Corsica, France), near the STARESO oceanographic station between 2002 and 2004. Samples were taken at a reference site (depth 10 m) followed by our laboratory since the 1970s, as well as along a permanent transect (7 to 35 m depth). At the level of a *Posidonia* shoot, particular attention was given to the spatio-temporal evolution of the structure of the epiphytic community. Specific composition of sessile fauna (mainly Bryozoa) was established at 7, 10, 15, 20, 25 and 30 m depth (Lepoint et al. 2014a). Temporal dynamics and trophic ecology of the dominant species *Electra posidoniae* was studied using seasonal sampling and isotopic approach (Lepoint et al. 2014b). Finally, we have measured experimentally the nitrogen uptake by different epiphytic macroalgae components (Lepoint et al. 2007). Complementary information about the epiphytic community of *Posidonia oceanica* in Revellata Bay may be also found in, for example, Dalla Via et al. 1998; Jacquemart and Demoulin 2006; Lepoint et al. 1999; Michel et al. 2015; Pête et al. 2015.

## Sampling methods

### Study extent

All sampling events (n = 103) and measurements were performed in Revellata Bay (Calvi, Corsica, France), near the marine research centre of STARESO (42°35'N, °43'E) (University of Liège) along the same permanent transects. Sampling encompassed one complete seasonal cycle and the 7 to 30 m depth range occupied by *Posidonia oceanica* shoots in Revellata Bay (see above).

### Sampling description

Seagrass shoots were collected in triplicate in November 2002, March, June, September and November 2003 along the same permanent transects set at 7, 10, 15, 20, 25 and 30 m depth. Each sample is constituted of a single replicate gathering three shoots of *Posidonia oceanica*.

### Quality control

To determine sample size, previous work was performed to determine the number of shoots that accumulates at least the 75% of the leaf epiphyte bryozoan species. For publication on GBIF portal, synonymies were matched against the authoritative, expert-driven World Register of Marine Species (WoRMS) and corrected compared to Lepoint et al. (2014a).

### Step description

The shoots were immediately frozen, then conserved in 4% formalin diluted in seawater. Identifications of species settled on leaves and scales (i.e. dead petioles remaining after leaf abscission) under a stereomicroscope (Stemi 2000, Zeiss) were done to the lowest systematic level using keys for Bryozoa (Prenant and Bobin 1966; Zabala and Maluquer 1988), and the works of Balduzzi et al. (1991), Gautier (1961) and Hayward and McKinney (2002). Colonies on scales were not counted and data are presented only as occurrence. All the colonies found on the two sides of leaves were counted. Colonies counted were reported per shoot and per metre square, accounting the average *Posidonia oceanica* shoot number per metre square, measured at each sampling depth. This parameter was measured monthly using a quadrate with an area of 0.1 m^2^ randomly set in the meadow. The shoot density did not show any significant variation during the duration of this study. Bathymetric variability of this parameter in Revellata Bay may be found in Gobert et al. (2003).

## Geographic coverage

### Description

Revellata Bay is a part of Calvi Bay and lies in the western Mediterranean, on the northwestern coast of Corsica (42°35'N, 8°45'E). Its western limit is Punta Revellata Cape, and its eastern limit is Punta San Francesco Cape. The STARESO (STAtion de REcherches Sous-marines et Océanographiques) research station (University of Liège) is located on Punta Revellata, at the western border of the bay. Salinity of the water of Calvi Bay is approximately 38‰, and is relatively invariant throughout the year. Temperature of water varies between minima of 12°C (February) and maxima of 26°C (August), with a notable vertical thermal stratification from May to September. Amplitude of tidal variation is weak. Nutrient concentrations (N, P) and particle load in the water column are typically low and characteristic of oligotrophic areas (Lepoint et al. 2004). *Posidonia oceanica* meadows cover approximately 50% of the area of the bay, and reach depths of nearly 40 m. Meadows show, in most places, a continuous extension, but local erosion (“intermattes”) occurs (Abadie et al. 2015). The vast majority of meadows grow on soft bottoms, but they seldom colonize rocky substrates. Meadows of Calvi Bay are relatively dense, and show an important foliar biomass and production despite the oligotrophic character of the area (Gobert et al. 2003).

### Coordinates

Latitude between 42.5799 and 42.5801; longitude between 8.7285 and 8.7245.

## Taxonomic coverage

### Description

The dataset includes 54 species of cheilostome Bryozoa, belonging to 25 different families.

### Taxa included

A full list of taxa included in this dataset is given in Table 1.

**Table 1.** List of taxa included in the dataset.

Rank	Scientific name
Kingdom	Animalia
Phylum	Bryozoa
Class	Gymnolaemata
Order	Cheilostomatida
Family	Aeteidae, Beaniidae, Bitectiporidae, Calloporidae, Candidae, Celleporidae, Chlidoniidae, Chorizoporidae, Cribrilinidae, Electridae, Epistomiidae, Escharinidae, Exochellidae, Flustridae, Haplopomidae, Lacernidae, Margarettidae, Microporellidae, Phidoloporidae, Romancheinidae, Savignyellidae, Schizoporellidae, Smittinidae, Umbonulidae, Watersiporidae
Species	*Aetea lepadiformis*, *Aetea truncata*, *Arthropoma cecilii*, *Beania hirtissima*, *Beania mirabilis*, *Beania robusta*, *Caberea boryi*, *Callopora lineata*, *Cellepora pumicosa*, *Celleporina caliciformis*, *Celleporina caminata*, *Celleporina decipiens*, *Chartella papyrea*, *Chlidonia pyriformis*, *Chorizopora brongniartii*, *Collarina balzaci*, *Copidozoum tenuirostre*, *Cradoscrupocellaria reptans*, *Electra posidoniae*, *Escharella rylandi*, *Escharina vulgaris*, *Escharoides coccinea*, *Escharoides mamillata*, *Fenestrulina joannae*, *Fenestrulina malusii*, *Figularia figularis*, *Haplopoma graniferum*, *Haplopoma impressum*, *Hincksina flustroides*, *Margaretta cereoides*, *Membraniporella nitida*, *Microporella ciliata*, *Parasmittina raigii*, *Parasmittina tropica*, *Prenantia cheilostoma*, *Puellina gattyae*, *Puellina hincksi*, *Puellina innominata*, *Puellina pedunculata*, *Savignyella lafontii*, *Schizobrachiella sanguinea*, Schizomavella (Calvetomavella) discoidea, Schizomavella (Schizomavella) auriculata, Schizomavella (Schizomavella) cornuta, Schizomavella (Schizomavella) hastata, *Schizoporella dunkeri*, *Schizotheca fissa*, *Scrupocellaria aegeensis*, *Scrupocellaria delilii*, *Scrupocellaria scrupea*, *Scrupocellaria scruposa*, *Synnotum aegyptiacum*, *Turbicellepora magnicostata*, *Umbonula ovicellata*, *Watersipora cucullata*

## Temporal coverage

### Data range

01 Nov 2002 – 31 Dec 2003.

### Usage rights

This dataset is under a Creative Commons Public domain CC0 license.

### Data resources

Data package title:

### Epiphytic Bryozoa of *Posidonia oceanica* leaves and scales. v1.0.

Resource link: http://ipt.biodiversity.be/resource?r=ulg_bryozoa&v=1.0

Alternative identifiers: doi: 10.15468/78vsgm

Data format: Darwin Core Archive

Data set version: 1.0

Data set description

Number of data files in the data set: 2

File 1 name: event.txt

Data format: CSV

Description: This file gathers data concerning sampling events (n= 103) (12 columns, 104 lines) (Table 2)

**Table 2.** Structure of the sampling events file.

Column label	Column description
eventID	Identification of the sampling event (n=103) (this key is used in the occurrence/abundance file (see below)
eventDate	Sampling event date
locationID	Sampling time (not location) identification number (n=5)
samplingSizeValue	Sampling area (in m2) used to measure the number of *Posidonia oceanica* shoots per metre square. This value was used to express our abundance data in number of colony per metre square.
sampleSizeUnit	Unit used to express abundance (number of colony per metre square)
minimumDepthInMeters	Depth of sampling locations (metres)
decimalLatitude	Latitude of sampling location (decimal)
decimalLongitude	Longitude of sampling location (decimal)
waterBody	Name of sampled water area (Revellata Bay, Mediterranean Sea)
locality	Name of sampled locality (Calvi, Corsica, France)
countryCode	International code of country
samplingProtocol	Url link to the web site (open repository of Liège University) to access the original paper explaining the protocol used to obtain this data set

File 2 name: occurrence.txt

Data format: CSV

Description: This file gathers occurrence data (n = 1234) (14 columns, 1235 lines) and is linked to file 1 by event identifiers (eventID column) (Table 3).

**Table 3.** Structure of the occurrence/abundance file.

Column label	Column description
occurrenceID	Identification of the occurrence
eventID	Identification number of sampling event (cf. sampling events file, Table 2)
scientificName	Binominal scientific name
tKingdom	Kingdom of the occurrence
tPhylum	Phylum of the occurrence
tClass	Class of the occurrence
tOrder	Order of the occurrence
tFamily	Family of the occurrence
taxonRank	Taxon rank of the occurrence
occurrenceStatus	Occurrence status (presence/absence)
occurrenceRemarks	Localisation of the colony on the plant (on seagrass leaf or on seagrass scales)
organismQuantity	number of colonies per square metre
organismQuantityType	Nature of counted items (colonies for all species)
dynamicProperties	Remarks/number of ovicellated colonies

## Author contributions

GL conceived the sampling design, performed the sampling and performed identification of specimens. LNM and AH formatted and published the dataset. LNM, GL & AH wrote the paper.
